# Prevalence of Pseudohypoparathyroidism and Nonsurgical Hypoparathyroidism in Japan in 2017: A Nationwide Survey

**DOI:** 10.2188/jea.JE20220152

**Published:** 2023-11-05

**Authors:** Rieko Takatani, Takuo Kubota, Masanori Minagawa, Daisuke Inoue, Seiji Fukumoto, Keiichi Ozono, Yosikazu Nakamura

**Affiliations:** 1 Center for Preventive Medical Sciences, Chiba University, Chiba, Japan; 2 Department of Pediatrics, Osaka University Graduate School of Medicine, Osaka, Japan; 3 Division of Endocrinology, Chiba Children’s Hospital, Chiba, Japan; 4 Third Department of Medicine, Teikyo University Chiba Medical Center, Chiba, Japan; 5 Department of Molecular Endocrinology, Fujii Memorial Institute of Medical Sciences, Institute of Advanced Medical Sciences, Tokushima University, Tokushima, Japan; 6 Division of Public Health, Center for Community Medicine, Jichi Medical University, Tochigi, Japan

**Keywords:** hypoparathyroidism, pseudohypoparathyroidism, nationwide survey, prevalence, Japan

## Abstract

**Background:**

Pseudohypoparathyroidism (PHP) and nonsurgical hypoparathyroidism (NS-HypoPT) are rare diseases with hypocalcemia, hyperphosphatemia, and high and low parathyroid hormone levels, respectively. In Japan, over 20 years have passed since the last survey on these diseases. We carried out a nationwide cross-sectional survey to estimate the prevalence of these diseases in 2018.

**Methods:**

We conducted a nationwide mail-based survey targeting hospitals in 2018. From a total of 13,156 departments throughout Japan, including internal medicine, pediatrics, neurology, and psychiatry, 3,501 (27%) departments were selected using a stratified random sampling method. We asked each included department to report the number of patients with PHP and NS-HypoPT in 2017.

**Results:**

The overall survey response rate was 52.0% (1,807 departments). The estimated number of patients with PHP and NS-HypoPT was 1,484 (95% confidence interval [CI], 1,143–1,825) and 2,304 (95% CI, 1,189–3,419), respectively; the prevalence per 100,000 population was 1.2 and 1.8, respectively.

**Conclusion:**

In this study, we generated estimates of the national prevalence of PHP and NS-HypoPT in Japan during 2017, which were found to be higher than those previously reported.

## INTRODUCTION

Pseudohypoparathyroidism (PHP) is defined as target organ resistance to parathyroid hormone (PTH), which results in hypocalcemia and hyperphosphatemia. PHP is clinically divided into PHP1A with Albright hereditary osteodystrophy (AHO), characterized by short stature, round face, obesity, brachydactyly, and heterotopic ossification, and PHP1B without AHO.^1^ PHP is caused by molecular defects that impair hormonal signaling via receptors that are coupled, through the alpha subunit of the stimulatory G protein (Gsalpha), to activation of adenylyl cyclase.^2^ Pseudopseudohypoparathyroidism (PPHP), progressive osseous heteroplasia (POH), and acrodysostosis are also disorders with impairments in the PTH and/or PTHrP cAMP-mediated pathway.^3^ These are very rare diseases, and the actual prevalence is largely unknown.^2^^–^^4^

Hypoparathyroidism is characterized by hypocalcemia owing to insufficient PTH secretion. Other than post-surgical hypoparathyroidism, idiopathic hypoparathyroidism was most frequent, even though a number of genetic causes of impaired PTH secretion have been identified. Additionally, there are many cases of unknown etiology.^5^

PHP and nonsurgical hypoparathyroidism (NS-HypoPT) are rare and are categorized as intractable diseases by the Ministry of Health, Labour and Welfare in Japan. An epidemiological survey on PHP and NS-HypoPT was conducted in Japan during 1997.^6^ The diagnostic criteria at that time are shown in eTable 1. Since then, molecular diagnostic methods have progressed and awareness of the disease has increased.^7^^,^^8^ For these reasons, we believe that reassessment of the epidemiological information is needed. The Research Committee on Epidemiology of Intractable Diseases (Chairperson: Yosikazu Nakamura) and the Hormone Receptor Abnormality Research Committee (Chairperson: Takashi Akamizu), sponsored by the Ministry of Health, Labour and Welfare of the Japanese government, jointly conducted a nationwide survey of PHP and NS-HypoPT in Japan during 2018.

The purpose of the present study was to determine the current number of patients with PHP and PHP-related diseases, as well as NS-HypoPT, in Japan, and to determine their clinical and epidemiological characteristics. In this report, we discuss only the prevalence from the first survey.

## METHODS

This nationwide survey was carried out using a protocol for epidemiological research on intractable diseases. This protocol was created by the study group of Epidemiological Research of Intractable Diseases Japan and was developed based on the concept that patients with intractable diseases tend to go to larger hospitals. Therefore, in this method, the target facilities were extracted by stratification according to the scale of the hospital and the clinical department, and the extraction rate was changed for each stratum to extract the survey facilities.^6^^,^^9^

Target diseases examined in the survey were PHP, PPHP, POH, acrodysostosis, and NS-HypoPT. The target clinical departments in the previous survey were pediatrics, neurology, internal medicine, and endocrinology. However, given the fact that endocrinology was not incorporated into the list of clinical departments in this study, the four clinical departments in the present survey included pediatrics, internal medicine, neurology, and neuropsychiatry.

The survey period was the full calendar year in 2017. The selection rate was 100% for hospitals with 500 beds or more and university hospitals, 80% for hospitals with 400 to 499 beds, 40% for hospitals with 300 to 399 beds, 20% for hospitals with 200 to 299 beds, and 10% for hospitals with 100 to 199 beds; only 5% of hospitals with fewer than 100 beds were selected at random. In addition to the four abovementioned departments, we designated pediatric specialty facilities as a special department. We then sent a questionnaire with diagnostic guidelines to all selected study departments (Table 1).

**Table 1.** Diagnostic guidelines for PHP, PPHP, POH, acrodysostosis, and NS-HypoPT for the nationwide epidemiologic survey

**PHP**		
	Definitive diagnosis had to satisfy 1 and 2:	
		1. Having PTH resistance
		2. Not having vitamin D deficiency
**PPHP**		
	Definitive diagnosis had to satisfy 1 and 2:	
		1. Having AHO
		2. Not having PTH resistance
**POH**		
	Definitive diagnosis had to satisfy 1 and 2:	
		1. Having ectopic ossification that was progressive and extended deep into connective tissue
		2. Having up to two features of AHO
**Acrodysostosis**		
	Definitive diagnosis had to satisfy 1 to 3:	
		1. Having characteristic facial features
		2. Having nasal hypoplasia
		3. Having brachydactyly
**Hypoparathyroidism (excluding secondary)^a^**		
	Definitive diagnosis had to satisfy 1 and 2:	
		1. Having hypocalcemia due to decreased secretion of parathyroid hormone
		2. Not having CKD
	<Exclusions>	
		① Secondary hypoparathyroidism (after cervical surgery, irradiation, malignant tumor infiltration, granulomatous disease, hemochromatosis, Wilson’s disease, and maternal primary hyperparathyroidism [newborn/transient])
		② Improvement of hypocalcemia after magnesium supplementation

AHO, Albright hereditary osteodystrophy; CKD, chronic kidney disease; NS-HypoPT, nonsurgical hypoparathyroidism; PHP, pseudohypoparathyroidism; POH, progressive osseous heteroplasia; PPHP, pseudopseudohypoparathyroidism; PTH, parathyroid hormone.

^a^In this study, hypoparathyroidism (excluding secondary) is considered the same as NS-hypoPT.

As a first survey, in February 2018, we inquired about the presence of patients with the diseases of interest and the number of cases in hospitals that were extracted using the above method. In August 2018, we submitted a second request to complete the first survey to hospitals that did not respond to our initial request.

During a secondary survey in October 2018, we distributed an individual survey form to facilities that reported having patients with the target diseases in the first survey.

The study protocol was approved by the Ethics Committee of Chiba University School of Medicine (approval number: 2940).

### Estimation of the number of patients

In consideration of the selection and response rate in the first survey, we estimated the total number of patients with PHP and NS-HypoPT. The total number of patients in Japan during the study period was calculated using the following formula:

$$\begin{array}{rl} \text{Estimated total number of patients} & =\text{number of reported patients}/(\text{sampling proportion}\times \text{response proportion})\\ & =\text{number of reported patients}\\ & \;/(\text{number of departments that responded}/\text{number of departments in Japan}). \end{array}$$

The numbers of patients for each stratum were summed. Subsequently, we calculated 95% confidence intervals assuming multinomial hypergeometric distribution.^10^^–^^14^

## RESULTS

Table 2 shows the number of sampled and responding departments, per medical department type, and the number of patients by department. From a total of 13,156 departments comprising internal medicine, pediatrics, neurology, and psychiatry throughout Japan, 3,501 (27%) study departments were selected at random. Of the departments that received the first questionnaire, 1,807 responded; the response rate was 52%.

**Table 2.** Numbers of sampled and responding departments in the nationwide survey

Department type	All departments	Sampled departments	%	responding departments	%
Internal Medicine	7,422	1,536	21	677	44
Pediatrics	2,649	856	32	649	76
Neurology	2,009	713	35	347	49
Psychiatry	1,076	396	37	134	34

Overall	13,156	3,501	27	1,807	52

The responding departments reported 478 patients with PHP and 704 patients with NS-HypoPT who visited hospitals in 2017 (Table 3). The details of patients with PHP were: 252 (53%) patients from university hospitals, 112 (23%) from hospitals with 500 beds or more, 43 (9%) from hospitals with 400 to 409 beds, and 35 (8%) from pediatric specialty facilities. There were 36 (7%) patients from other facilities. The details of patients with NS-HypoPT were: 330 (47%) patients from university hospitals, 190 (27%) from hospitals with 500 beds or more, 71 (10%) from hospitals with 400 to 409 beds, and 51 (7%) from pediatric specialty facilities. There were 62 (9%) patients from other facilities. In this way, many of these patients were reported from university hospitals or larger hospitals. For other diseases, 19 patients were reported with PPHP, 5 with POH, and 7 with acrodysostosis.

**Table 3.** Number of patients reported by departments

	department				total

	Internal Medicine	Pediatrics	Neurology	Psychiatry	
PHP	272	198	8	0	478
NS-HypoPT	540	156	7	1	704

NS-HypoPT, nonsurgical hypoparathyroidism; PHP, pseudohypoparathyroidism.

Table 4 and Table 5 show the numbers of patients with PHP and NS-HypoPT, estimated statistically on the basis of the values obtained from the extracted samples. The number of patients with PHP was estimated to be 1,480, whereas the number with NS-hypoPT was estimated as 2,300. From these estimates and the estimated 2017 Japanese population of 124.48 million, the prevalence of PHP and NS-HypoPT was 1.2 and 1.8 per 100,000 inhabitants, respectively.

**Table 4.** Estimated numbers of patients with pseudohypoparathyroidism (PHP) in Japan in 2017

Department type	Estimated number of patients	95% confidence interval
Internal Medicine	1,191	852–1,530
Pediatrics	276	245–308
Neurology	17	7–27
Psychiatry	0	

total^a^	1,480	1,140–1,830

^a^Totals were calculated with significant figures up to the tenth decimal place and rounded to the nearest whole number.

**Table 5.** Estimated numbers of patients with nonsurgical hypoparathyroidism (NS-hypoPT) in Japan in 2017

Department type	Estimated number of patients	95% confidence interval
Internal Medicine	2,059	944–3,175
Pediatrics	226	191–261
Neurology	16	5–27
Psychiatry	3	0–7

total^a^	2,300	1,190–3,420

^a^Totals were calculated with significant figures up to the tenth decimal place and rounded to the nearest whole number.

The distribution by disease, age, and sex of patients from the second survey is shown in Table 6. Of the 363 cases reported with NS-HypoPT, 195 (54%) were male, whereas 105 (57%) of the 241 cases reported with PHP were female. NS-hypoPT occurred at a similar rate across all age groups while PHP was concentrated in patients under the age of 60 years. Table 7 presents details of the clinical diagnosis of NS-hypoPH. Idiopathic hypoparathyroidism and DiGeorge syndrome accounted for the majority of patients.

**Table 6.** Age distribution by disease and sex

Age, years	NS-hypoPT			PHP		
	
	male	female	total	male	female	total
0–9	20 (10)	21 (13)	41 (11)	12 (11)	8 (5)	20 (8)
10–19	26 (13)	29 (18)	55 (15)	37 (35)	54 (40)	91 (38)
20–29	30 (15)	9 (6)	39 (11)	26 (25)	21 (15)	47 (20)
30–39	17 (9)	21 (13)	38 (10)	13 (12)	19 (14)	32 (13)
40–49	23 (12)	24 (15)	47 (13)	7 (7)	19 (14)	26 (11)
50–59	25 (13)	21 (13)	46 (13)	7 (7)	10 (7)	17 (7)
60–69	29 (15)	17 (11)	47 (13)	1 (1)	4 (3)	5 (2)
70–79	18 (9)	13 (8)	31 (9)		1 (1)	1 (0)
≥80	7 (4)	3 (2)	20 (5)	2 (2)		2 (1)
total	195 (100)	158 (100)	363 (100)	105 (100)	136 (100)	241 (100)

NS-hypoPT, nonsurgical hypoparathyroidism; PHP, pseudohypoparathyroidism.

**Table 7.** Number of cases with each disease type among cases with nonsurgical hypoparathyroidism (NS-hypoPT) in Japan

Diseases	*n*	%
Idiopathic hypoparathyroidism	241	68.3
DiGeorge syndrome	69	19.5
HDR syndrome	17	4.8
Diseases associated with CaSR	17	4.8
Mitochondrial disorders	2	0.6
Others	7	2.0

Total	353	

CaSR, calcium-sensing receptor; HDR, Hypoparathyroidism, Deafness, and Renal Dysplasia.

## DISCUSSION

In this study, we estimated that the prevalence rate per 100,000 individuals for PHP and NS-HypoPT was 1.2 and 1.8, respectively. The present study was preceded by a study in 1998 on PHP and NS-HypoPT. Nakamura et al reported the results of a previous survey, with a prevalence per 100,000 for PHP and NS-HypoPT of 0.34 and 0.72, respectively.^6^ Compared with results of the previous survey, the prevalence per 100,000 inhabitants was higher for both PHP and NS-HypoPT in our study. The difference in department selection versus the previous study may have affected results. Although psychiatry, which was not included in the previous survey, was included in the present survey, almost no cases were reported; therefore, we considered that this inclusion had no effect on the prevalence (eTable 3 and eTable 4). In this study, four clinical departments were targeted and the possibility of duplication cannot be denied. However, identical cases were not observed in the personal survey table of the secondary survey; thus, we assumed no significant impact. Consistent with the findings of the previous survey, Ns-hypoPT patients were predominantly male, while PHP patients were largely female (Table 6). Age distribution was similar to that in the previous survey, with PHP more prevalent than NS-hypoPT in the younger generation and NS-hypoPT relatively common among men aged 50 years and above.

In actuality, the reason for the increase in the number of patients with both diseases is not clear. This may be related to problems of recognition, as well as a true increase. The diagnostic rate may have increased because awareness about PHP and NS-HypoPT has increased and molecular genetic diagnostic methods have advanced.^15^^,^^16^ PHP may be suspected in patients who present with AHO and hypothyroidism, and PTH resistance has been found before the onset of hypocalcemia.^15^ There have also been reports of cases in which DiGeorge syndrome was genetically diagnosed, as distinguished from other symptoms, and hypoparathyroidism was diagnosed as a result of close examination.^16^

There is little epidemiological information from other countries regarding either PHP or NS-HypoPT. In 2016, Underbjerg et al reported a prevalence of PHP was 1.1 per 100,000 in Denmark and Astor et al reported that the prevalence of PHP was 0.82 per 100,000 in Norway.^17^^,^^18^ Regarding NS-HypoPT, Underbjerg et al reported that the prevalence of NS-HypoPT was 2.3 per 100,000 in Denmark in 2015.^5^ In 2016, Astor et al reported that the prevalence of NS-HypoPT was 0.78 per 100,000 in Norway.^17^ The prevalence in our study was similar to that reported in Denmark in 2016 and 2015 for both PHP and NS-HypoPT (Table 8).

**Table 8.** Prevalence of pseudohypoparathyroidism (PHP) and nonsurgical hypoparathyroidism (NS-HypoPT)

PHP	Country	Reported year	Sampling method	Population	number of cases	Prevalence per 100,000	Reference
	This study	—	A nationwide mail survey	124,480,000	1,480^a^	1.2	—
	Norway	2016	Electronic hospital registry	4,985,870	41^b^	0.82	17
	Denmark	2016	National hospital patient registry	5,336,394	60^b^	1.1	18
	Japan	2000	A nationwide mail survey	125,000,000	430^a^	0.34	6

NS-hypoPT	Country	Reported year	Sampling method	Population	number of cases	Prevalence per 100,000	Reference

	This study	—	A nationwide mail survey	124,480,000	2,300^a^	1.8	—
	Norway	2016	Electronic hospital registry	4,985,870	151^b^	3	17
	Denmark	2015	National hospital patient registry	5,336,394	123^b^	2.3	5
	Japan	2000	A nationwide mail survey	125,000,000	900^a^	0.72	6

^a^Estimated total number of patients from a nationwide mail survey conducted in a part of hospitals in Japan.

^b^The total number of patients identified by the registry.

There are several limitations to this study. First, because patients in clinics with fewer than 20 beds were not included, the prevalence may be underestimated. Despite this, it is likely that patients with both diseases tend to be treated in large, highly specialized hospitals and were generally covered by our survey. Second, the estimated prevalence was calculated on the assumption that the prevalence of PHP and NS-HypoPT is the same in those hospitals that did not respond to our survey. Hospitals with no cases may not even reply. Therefore, hospitals that responded may be biased toward those that have patients with the diseases of interest. In internal medicine, cases from smaller hospitals with a low sampling rate affect the total estimated number of patients and widen the 95% confidence interval, undeniably influencing the overestimate (eTable 3 and eTable 4). From the above, we may have overestimated the number of patients. Finally, the diagnoses were completely dependent on the attending physicians and were not confirmed using biochemical data.

In conclusion, we determined the prevalence of PHP and NS-HypoPT in Japan in 2017. The overall prevalence rate per 100,000 individuals for PHP and NS-HypoPT was 1.2 and 1.8 in that year, respectively. These estimates are higher than those in 1997, suggesting increasing disease burden.

## SUPPLEMENTARY MATERIAL

eTable 1. Diagnostic guidelines of idiopathic hypoparathyroidism and pseudohypoparathyroidism for the nationwide epidemiologic survey, by the Hormone Receptor Abnormality Research Committee, Ministry of Health and Welfare

eTable 2. Numbers of sampled and responding departments in the nationwide survey

eTable 3. Number of cases with pseudohypoparathyroidism reported by departments

eTable 4. Number of cases with nonsurgical hypoparathyroidism reported by departments
