# Supplementary material for: Prevalence of Pseudohypoparathyroidism and Nonsurgical Hypoparathyroidism in Japan in 2017: A Nationwide Survey
Source: J Epidemiol. 2023 Nov 5;33(11):569–73. doi: 10.2188/jea.JE20220152 (PMC10518380; doi:10.2188/jea.JE20220152)
Supplement: Supplementary file 1 [file je-33-569-s001.pdf]

eTable 1. Diagnostic guidelines of idiopathic hypoparathyroidism and pseudohypoparathyroidism for the nationwide epidemiologic survey, by the Hormone Receptor Abnormality Research Committee, Ministry of Health and Welfare

1. Idiopathic hypoparathyroidism

    A. Low level of serum intact parathyroid hormone

        Serum intact parathyroid hormone <30 pg/mL 1)

    B. Hypocalcemia

        Serum calcium <8.5 mg/dL 2)

    C. Absence of decreased serum phosphate (increase or normal)

        Serum phosphate equal to or greater than 3.5 mg/dL (15 years of age or older)

        Serum phosphate equal to or greater than 4.5 mg/dL (<15 years of age or older)

    D. Absence of renal dysfunction

        Serum BUN equal to or less than 30 mg/dL

        or serum creatinine equal to or less than 2 mg/dL

    E. Not to be the results of other diseases (eg, hyperthyroidism), nor secondary symptoms of other diseases

2. Pseudohypoparathyroidism

    A. High level of serum intact parathyroid hormone

        Serum intact parathyroid hormone equal to or greater than 30 pg/mL 1)

    B. Hypocalcemia

        Serum calcium <8.5 mg/dL 2)

    C. Absence of decreased serum phosphate (increase or normal)

        Serum phosphate equal to or greater than 3.5 mg/dL (15 years of age or older)

        Serum phosphate equal to or greater than 4.5 mg/dL (<15 years of age or older)

    D. Absence of renal dysfunction

        Serum BUN equal to or less than 30 mg/dL

        or serum creatinine equal to or less than 2 mg/dL

    E. Not to be the Secondary symptoms of other diseases, such as renal tubule dysfunction

1) Immunoradiometric assay by Nussbum SR, et al. (Clin Chem 1987; 33: 1364)

2) In case of hypoalbuminemia (<4g/dL), hypocalcemia should be

    correct calcium=observed calcium + (4 - serum albumin (g/dL)) <8.5

    In institutes where ionized calcium is observed directly, hypocalcemia should be defined if the level is less than the lowest value of normal range at the institutes

Remarks	1	All the laboratory data should be fasting in the morning
	2	In the guidelines, b through e for idiopathic hypoparathyroidism and pseudohypoparathyroidism are identical. The item a divides the diagnosis between idiopathic hypoparathyroidism and pseudohypoparathyroidism. The nationwide survey requires all cases that satisfy the item b through e.
	3	The diagnosis of pseudohypoparathyroidism should depend not only on the serum parathyroid hormone but also on the results of Ellsworth-Howard test. Besides, even though such a patient exists that hypoparathyroidism with manifest renal dysfunction, the nationwide survey does not observe such a patient.

[a] In this study, idiopathic hypoparathyroidism is considered the same as NS-hypoPT.

eTable 2. Numbers of sampled and responding departments in the nationwide survey

Department type	Numbers of beds or hospital type	All departments	Sampled departments	%	responding departments	%
Internal Medicine	University hospitals	142	142	100	91	64
	>500 beds	298	298	100	147	49
	400–499 beds	355	283	80	137	48
	300–399 beds	622	247	40	101	41
	200–299 beds	945	189	20	60	32
	100–199 beds	2,480	248	10	91	37
	≤99 beds	2,580	129	5	50	39
	Subtotal	7,422	1536	21	677	44
Pediatrics	Specialized hospitals	35	35	100	23	66
	University hospitals	119	119	100	101	85
	>500 beds	212	212	100	170	80
	400–499 beds	230	184	80	142	77
	300–399 beds	348	139	40	108	78
	200–299 beds	325	65	20	42	65
	100–199 beds	660	66	10	36	55
	≤99 beds	720	36	5	27	75
	Subtotal	2,649	856	32	649	76
Neurology	University hospitals	103	103	100	70	68
	>500 beds	205	205	100	101	49
	400–499 beds	181	151	80	71	47
	300–399 beds	290	116	40	57	49
	200–299 beds	300	60	20	24	40
	100–199 beds	630	63	10	19	30
	≤99 beds	300	15	5	5	33
	Subtotal	2,009	713	35	347	49
Psychiatry	University hospitals	58	58	100	25	43
	>500 beds	83	83	100	32	39
	400–499 beds	103	82	80	28	34
	300–399 beds	187	74	40	23	31

	200–299 beds	285	57	20	15	26
	100–199 beds	280	38	10	9	24
	≤99 beds	80	4	5	2	50
	Subtotal	1,076	396	37	134	34
Overall		13,156	3501	27	1,807	52

eTable 3. Number of cases with pseudohypoparathyroidism reported by departments

Department type	Numbers of beds or hospital type	Estimated number	standard error	95% CI	
Internal Medicine	University hospitals	270	18	235	305
	>500 beds	97	17	64	131
	400–499 beds	60	20	20	99
	300–399 beds	68	28	13	123
	200–299 beds	47	26		98
	100–199 beds	82	70		219
	≤99 beds	568	150	274	862
	Subtotal	1,191	173	852	1,530
Pediatrics	Specialized hospitals	92	7	78	106
	University hospitals	74	6	62	85
	>500 beds	29	5	19	39
	400–499 beds	19	7	6	33
	300–399 beds	8	1	6	10
	200–299 beds	0			
	100–199 beds	0			
	≤99 beds	55	10	35	74
	Subtotal	276	16	245	308
Neurology	University hospitals	1	3		7
	>500 beds	10	3	4	16
	400–499 beds	5	3		11
	300–399 beds	0			
	200–299 beds	0			
	100–199 beds	0			
	≤99 beds	0			
	Subtotal	17	5	7	27
Psychiatry	University hospitals	0			
	>500 beds	0			
	400–499 beds	0			
	300–399 beds	0			

	200–299 beds	0			
	100–199 beds	0			
	≤99 beds	0			
	Subtotal	0			
Overall[a]		1,480	174	1,140	1,820

CI, confidence interval.

[a] The overall values for the estimated number of patients and 95% confidence intervals were calculated with significant figures up to the tenth decimal place and rounded to the nearest whole number.

eTable 4. Number of cases with nonsurgical hypoparathyroidism reported by departments

Department type	Numbers of beds or hospital type	Estimated number	standard error	95% CI	
Internal Medicine	University hospitals	421	53	317	525
	>500 beds	316	37	244	389
	400–499 beds	145	20	106	184
	300–399 beds	166	73	23	309
	200–299 beds	173	98		365
	100–199 beds	218	213		636
	≤99 beds	619	509		1,617
	Subtotal	2,059	569	944	3,175
Pediatrics	Specialized hospitals	71	5	61	80
	University hospitals	36	4	28	44
	>500 beds	19	4	12	27
	400–499 beds	6	4		14
	300–399 beds	15	10		35
	200–299 beds				
	100–199 beds				
	≤99 beds	78	12	54	101
	Subtotal	226	18	191	261
Neurology	University hospitals	0			
	>500 beds	8	4		16
	400–499 beds	8	4		15
	300–399 beds	0			
	200–299 beds	0			
	100–199 beds	0			
	≤99 beds	0			
	Subtotal	16	6	5	27
Psychiatry	University hospitals	0			
	>500 beds	3	2		7

	400–499 beds	0			
	300–399 beds	0			
	200–299 beds	0			
	100–199 beds	0			
	≤99 beds	0			
	Subtotal	3	2		7
Overall[a]		2,300	569	1,190	3,420

CI, confidence interval.

[a] The overall values for the estimated number of patients and 95% confidence intervals are shown with significant figures up to the tenth place and rounded off to the nearest one.
